# Lung Diffusion in a 14-Day Swimming Altitude Training Camp at 1850 Meters

**DOI:** 10.3390/ijerph17103501

**Published:** 2020-05-17

**Authors:** Iker García, Franchek Drobnic, Teresa Galera, Victoria Pons, Ginés Viscor

**Affiliations:** 1 Secció de Fisiologia, Departament de Biologia Cel·lular, Fisiologia i Immunologia, Facultat de Biologia, Universitat de Barcelona, Av. Diagonal, 643, E-08028 Barcelona, Spain; ikergarciaalday@gmail.com (I.G.); teresa.galera@gmail.com (T.G.); 2 Centre d’Alt Rendiment (CAR) de Sant Cugat, Av. Alcalde Barnils s/n, E-08173 Sant Cugat del Vallés, 08174 Barcelona, Spain; vpons@car.edu; 3 Medical Services Shenhua Greenland FC, Shanghai 201315, China; docdrobnic@gmail.com

**Keywords:** pulmonary diffusing capacity, DL_CO_, altitude training, swimming, SIPO

## Abstract

Swimming exercise at sea level causes a transient decrease in lung diffusing capacity for carbon monoxide (DL_CO_). The exposure to hypobaric hypoxia can affect lung gas exchange, and hypoxic pulmonary vasoconstriction may elicit pulmonary oedema. The purpose of this study is to evaluate whether there are changes in DL_CO_ during a 14-day altitude training camp (1850 m) in elite swimmers and the acute effects of a combined training session of swimming in moderate hypoxia and 44-min cycling in acute normobaric severe hypoxia (3000 m). Participants were eight international level swimmers (5 females and 3 males; 17–24 years old; 173.5 ± 5.5 cm; 64.4 ± 5.3 kg) with a training volume of 80 km per week. The single-breath method was used to measure the changes in DL_CO_ and functional gas exchange parameters. No changes in DL_CO_ after a 14-day altitude training camp at 1850 m were detected but a decrease in alveolar volume (VA; 7.13 ± 1.61 vs. 6.50 ± 1.59 L; *p* = 0.005; d = 0.396) and an increase in the transfer coefficient of the lung for carbon monoxide (K_CO_; 6.23 ± 1.03 vs. 6.83 ± 1.31 mL·min^−1^·mmHg^−1^·L^−1^; *p* = 0.038; d = 0.509) after the altitude camp were observed. During the acute hypoxia combined session, there were no changes in DL_CO_ after swimming training at 1850 m, but there was a decrease in DL_CO_ after cycling at a simulated altitude of 3000 m (40.6 ± 10.8 vs. 36.8 ± 11.2 mL·min^−1^·mmHg^−1^; *p* = 0.044; d = 0.341). A training camp at moderate altitude did not alter pulmonary diffusing capacity in elite swimmers, although a cycling session at a higher simulated altitude caused a certain degree of impairment of the alveolar–capillary gas exchange.

## 1. Introduction

**Figure 1:**
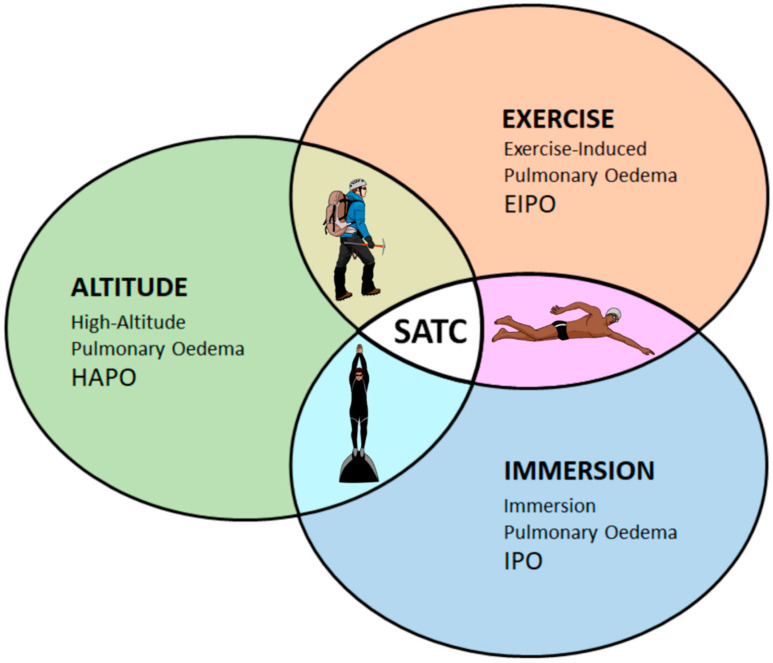
Schematic representation of external stimuli for the development of environmental-induced pulmonary oedema in healthy individuals. At high altitude, mountaineers are exposed to the effects of hypoxic pulmonary vasoconstriction, and increased pulmonary blood flow during exercise, thus developing the risk for high-altitude pulmonary oedema. Breath-holding divers suffer the simultaneous effects of hypoxia, central blood-shift, and chest compression. Swimmers experience hemodynamic changes in pulmonary circulation during exercise, due to regular breath-holding pattern and chest compression. SATC (swimming altitude training camp) combines all three forms of environmental stress that can elicit sub-clinical pulmonary oedema. Based on Marabotti et al. [2].

Swimming increases mechanical stress on the pulmonary system since it combines water immersion, exercise and breath-holding periods, leading to subtle changes in the permeability of the lungs [1]. Altitude exposure can be an added physiological challenge on the pulmonary system, raising the susceptibility to pulmonary oedema in combination with exercise, water immersion and hypoxia (Figure 1) [2].

Endurance training at sea level triggers significant physiological adjustments among cardiovascular, musculoskeletal and haematological systems, but the structural and functional properties of the lung and airways do not change in response to training in land-based sports. However, aquatic sports such as swimming [3,4,5], artistic swimming [6], and apnoeic diving [7] elicit improvements of the lung capacity and diffusion. During exercise, swimmers are exposed to a reduced frequency breathing against the hydrostatic forces produced by the water, which requires larger inspiration, and it can mimic intermittent hypoxic training where hypercapnia and hypoxia occur [8]. 

The diffusion capacity for carbon monoxide (DL_CO_) describes the conductance of gas from the alveolar air to the capillaries and provides a measure of gas transfer in the lungs [9]. Training camps at moderate altitudes are typically utilized among coaches to improve performance at sea level [10]. Different modalities of altitude training have been proposed [11] although Living High–Training High (LHTH) protocol is still the most feasible modality among swimmers due to the logistic limitation to locate an adequate swimming pool at a low altitude closer to the altitude training facility. A possible increase in lung diffusing capacity after altitude exposure would be a favourable adaptation in swimmers by allowing them to maintain gas exchange efficiency at a lower energy cost of breathing [12]. While a superior lung diffusing capacity has been reported in native highlanders [12], at the best of our knowledge, it remains unknown whether a short-term altitude training camp can achieve similar enhancements in pulmonary gas exchange in elite swimmers. Modifications of lung diffusing capacity in lowlanders sojourning at altitude still remain unclear with an overall pattern of minimal changes or a slight transient initial increase or decrease [13,14].

On the other hand, strenuous exercise at sea level may cause transient mild interstitial oedema [15,16], influenced by an increased pulmonary capillary pressure [17], which eventually causes leakage of fluid into the lung interstitium [18]. Accordingly, lung diffusing capacity measured after strenuous exercise at sea level has been reported to be decreased [19,20,21] which, normally, may not be an inconvenience in the highly-developed lungs from elite swimmers (unpublished) but it still remains unknown whether the exposure to swimming altitude training camps (SATCs) may impair the alveolar–capillary gas exchange. Pulmonary oedema has been related to outdoor swimming and also to high altitude exposure with different specific denominations: *high-altitude pulmonary oedema (HAPO)* and *swimming-induced pulmonary oedema (SIPO)*, although the occurrence rate of lung interstitial oedema under both conditions remains unclear [18,22,23]. The combination of hypoxic pulmonary vasoconstriction, basal hyperventilation at altitude and intense swimming may stress the respiratory system, limiting its functional capacity [24]. Subjects with higher FVC and VA showed more resistance to HAPO than counterparts suggesting that the compensatory rise in ventilation and pulmonary circulation could play a crucial role under environmental stress [25].

The question faced in this study is whether recurrent minor lung injuries could lead to long-term pulmonary deterioration. This could be of great practical importance, given the large number of competitive swimmers who are exposed themselves to repetitive environmental stress during altitude training camps [26]. Altitude training camps are extensively utilized by elite swimming coaches, but the possible modifications of the alveolar–capillary exchange are still unknown. Thus, the aim of this study is to evaluate the possible changes in lung diffusion capacity after 14 days of an altitude training camp in elite swimmers and the acute effects of a combined training session of swimming at 1850 m of real altitude (hypobaric hypoxia) and cycling at 3000 m simulated altitude (normobaric hypoxia). We hypothesize that a 14-day altitude training camp will increase lung diffusion capacity in swimmers, while the possible positive or detrimental effect of an acute combined training session is unknown.

## 2. Materials and Methods 

### 2.1. Subjects

The participants were eight international level swimmers (5 females and 3 males) aged 17 to 24 years old. The average FINA (Fédération Internationale de Natation) points in their best event were 827 FINA points at the time of the study. The athlete’s swim specialties were 6 middle-distance (200 or 400 m) and 2 long-distance (800 and 1500 m) swimmers. None of them suffered from asthma.

### 2.2. Experimental Design

The Altitude Training Camp was placed in the Centre National d’Entraînement en Altitude de Font-Romeu (France). The training schedule was 10 swimming sessions and 6 dry-land sessions per week throughout 30 h of training per week. The altitude training protocol utilized was the LHTH modality that is routinely used by the Royal Spanish Swimming Federation, where they lived at 1850 m during the whole altitude training camp. The weekly training volume was 80 kilometres, and the volume of swimming per session was 7000 to 9000 m. To evaluate the changes in lung diffusion induced by the altitude training camp, DL_CO_ was measured at rest 72 h after arrival to avoid the acute changes in diffusing capacity associated with hyperventilation produced as an immediate response to altitude exposure; then, a second DL_CO_ measurement was taken at rest the last day of the camp. The participants realized two measures before the beginning of the study to be familiar with the procedure. For the study of acute changes in lung diffusing capacity, the system was placed in a corner 10 m away from the pool where the swimmers did the training session to perform the measures before and after swimming training. Measurements were performed less than 5 min before the start of the warm-up and less than 5 min after the end of the training session. 

The combined training Session was performed on the 10th day of the Altitude Training Camp. The protocol consisted of a first swimming training that covered 7500 m of moderate-intensity at a moderate altitude (1850 m). This was followed by a cycling exercise performed in a normobaric hypoxic room (3000 m) lasting 44 min. The system was moved out of the normobaric hypoxic chamber to evaluate the lung diffusion capacity at the end of the cycling session. Cycling exercise intensity alternated 8 min of moderate-intensity with 3 min of low-intensity until completing 4 series. All the participants were familiar with this protocol; they performed similar cycling sessions in the normobaric chamber in previous altitude training camps, and they performed 6 similar cycling sessions during the 14-day altitude training camp.

### 2.3. DL_CO_ Measurements

The procedure which was used for obtaining the diffusing lung capacity parameters was the single-breath method by means of a computerized spirometer (Easy One Pro, ndd Medical Technologies, Zurich, Switzerland) attached to a gas mixture cylinder. This method involves measuring the uptake of CO from the lung over a short breath-holding period. The recommendations made in a recent joint statement by the American Thoracic Society (ATS) and the European Respiratory Society (ERS) were followed [27]. The results obtained in DL_CO_ were adjusted for the change in PAo2 due to barometric pressure (DL_CO_ adj) and haemoglobin (Hb) concentration that was determined from a small blood sample obtained by venepuncture to adjust DL_CO_ to individual parameters before the beginning of the study and at the end of the study. The participants were placed in a seated position, with a mouthpiece and nose-clip in place throughout the test procedure. The test started with tidal breathing for 2–4 breaths until the subject felt comfortable with the mouthpiece. Then the DL_CO_ manoeuvre began with an unforced exhalation to residual volume (RV). At RV, the subject’s mouthpiece was connected to a source of test gas, and the subject inhaled rapidly to maximal inspiration. After that, the participant was asked to hold their breath for 10 s and then exhale completely without interruption in less than 4 s and to continue with a tidal breath to finish the test. All measures considered were “grade A”, as identified by the system [27], and DL_CO_ was corrected to the individual. A maximum of 3 trials was performed. At least 4 min was allowed between trials to ensure adequate washout of the gases. The test gas mixture used to evaluate the pulmonary function and diffusion capacity was 0.3% of CO, 11% of a tracer inert gas (He) used to measure alveolar volume (VA), and the initial alveolar CO, and a mixture of 20.9% of O_2_ balanced with N_2_. In addition, the transfer coefficient of the lung for carbon monoxide (K_CO_), total lung capacity (TLC), vital capacity inspired (VC_IN_), and residual volume (RV) were calculated.

### 2.4. Ethical Considerations

All procedures were in accordance with the ethical standards of the Clinical Research Ethics Committee at the *Direcció General de l’Esport* of the Catalonian Sports Council (05-2020-CEICEGC). The study followed the principles of the Declaration of Helsinki for human experimentation. Informed consent was obtained to perform any type of test and evaluation procedures from the participants or their parents as it is mandatory for the athletes pertaining to the national swimming team and training along the entire season at the National Olympic Training Center of Sant Cugat del Valles.

### 2.5. Statistical Analysis

The results are reported as mean values ± standard deviation (SD). Differences in pulmonary parameters between pre- and post-altitude training camp were assessed using a paired sample *t*-test. Differences in pulmonary parameters during the acute combined session (pre- to mid- to post-training) were analyzed using one-way repeated-measures analysis of variance (ANOVA). Effect size (Cohen’s d) was calculated to estimate the magnitude of the difference between group means, with d = 0.2, 0.5, and 0.8 reflecting small, medium, and large effect sizes, respectively [28]. The level of significance was set at *p* < 0.05 for all statistical comparisons. The software package used for the statistical analysis was SPSS ver26 (IBM SPSS Statistics, Armonk, NY, USA).

## 3. Results

### 3.1. Anthropometrical Parameters 

Table 1 shows the average values and SD of physical and anthropometrical data of our sample of elite swimmers. The participants show similar anthropometrical values compared to those found in elite open water swimmers [29] and young amateur swimmers [30]. Spirometric values were higher than predicted for their age and height in forced vital capacity (FVC) and forced expiratory volume in 1-s (FEV1), both females (FVC: 108 ± 10% and FEV1: 107 ± 7%; Table 1) and males (FVC: 114 ± 18% and FEV1: 108 ± 16%; Table 1).

### 3.2. Changes in Lung Capacity and Function After 14-Day Altitude Training Camp at 1850 m

Table 2 shows the changes recorded in the different parameters of pulmonary function among the 14-day altitude training camp as mean ± SD. There were no changes in DL_CO_ adj after 14 days of altitude training camp in our sample of elite swimmers. However there were significant decreases in VA (7.13 ± 1.61 vs. 6.50 ± 1.59 L, *p*-value = 0.005; d = 0.396; Table 2) and TLC (7.28 ± 1.61 vs. 6.65 ± 1.59 L, *p*-value = 0.005; d = 0.396; Table 2) and a significant improvement in K_CO_ (6.23 ± 1.03 vs. 6.83 ± 1.31 mL·min^−1^·mmHg^−1^·L^−1^, *p*-value = 0.038; d = 0.509; Table 2).

### 3.3. Changes in Lung Capacity and Function After a Combined Session of Swimming at 1850 m and Cycling at 3000 m

When measuring DL_CO_ within a combined session of swimming at moderate altitude (1850 m) and cycling at high-altitude (3000 m) there were significant differences in DL_CO_ from post-swimming compared to post-cycling (40.6 ± 10.8 vs. 36.8 ± 11.5 mL·min^−1^·mmHg^−1^, *p*-value = 0.044; d = 0.341; Table 3) and K_CO_ was also slightly, but no significantly decreased after cycling (6.34 ± 1.00 vs. 6.27 ± 1.16 vs. 6.17 ± 1.13 mL·min^−1^·mmHg^−1^·L^−1^, *p*-value = 0.053; d = 0.087; Table 3). Also, there was a decrease in RV from basal to post-cycling (2.37 ± 0.63 vs. 2.16 ± 0.65 vs. 1.78 ± 0.59 L, *p*-value = 0.001; d = 0.966; Table 3).

## 4. Discussion

This study shows that elite swimmers with previous experience in altitude training and extremely high basal values in DL_CO_ do not suffer any change in lung diffusing capacity after 14 days of altitude training camp. During acclimatization to altitude, organs that experience high O_2_ demands, such as skeletal and cardiac muscle, are downregulated as a way of minimising hypoxic tissue injury and maximising the efficiency of O_2_ utilization [31,32]. At the same time, organs involved in O_2_ uptake, such as blood and the lungs, the alveolar–capillary gas exchange increases their capacities [33]. However, because these responses are limited, aerobic performance is impaired by a progressive reduction in maximum O_2_ uptake (VO_2_max) as altitude increases.

Our results show that DL_CO_ remains unaffected, although there is a decrease in VA and TLC, and an increase in K_CO_, probably produced as a compensatory adaptive mechanism to keep DL_CO_ levels stable against a reduced alveolar expansion or alveolar damage [34]. The decrease in VA may be a consequence of a decrease in right ventricular function and cardiac output after altitude acclimatization, which produces longer pulmonary capillary erythrocyte transit time, resulting in less alveolar/end capillary diffusion unbalance [35,36]. Lungs may also have an anti-oedematous response with marked alveolar vasoconstriction triggering a decrease in pulmonary blood capillary volume (V_C_) and an increase in membrane diffusive capacity (D_M_) [37] when facing hypobaric hypoxia exposure, and a slight increase in hemoglobin concentration at the end of the altitude stay could also contribute to the increase in K_CO_ after the altitude training camp.

A 14-day exposure to moderate altitude training camp may not provide ventilatory stimuli enough to modify pulmonary function, by inducing lung growth or altering alveolar–capillary gas exchange associated with SATC. Most of the studies evaluating changes in diffusing capacity has been performed on active mountaineers at high altitude, a condition that differs considerably from the situation faced by elite trained subjects exercising at moderate altitude. Different studies have been conducted to assess the effect of hypobaric hypoxia in lung diffusion properties, yielding conflicting results. Lung diffusing capacity for carbon monoxide (DL_CO_) has been reported to either increase or decrease after short- or long-term exposures to altitude. Faoro et al. [13] found that DL_CO_, K_CO_ and VA increased after acute exposure to moderate altitude (2250 m) of only 1 h. DL_CO_ was also significantly increased after 9 days at 5150 m [38] and after three weeks at 5400 m [39]. Martinot et al. [40] showed an increase in DL_CO_ from sea level to days 2–3 at 4300 m, but there was a decrease to the sea level values in VA and K_CO_ from days 2–3 to 7–8. In contrast, DL_CO_ did not change after acclimatization in 5050 m, although VA was decreased after 2 weeks of exposure [41]. Diffusing capacity also remained unchanged during a rapid ascent (1–3 days) in control subjects [42] and after a moderate stance (7–10 days) [43] at 4559 m. Senn et al. [14] also showed a slight decrease after a rapid ascent (1–2 d) to 4559 m, and subjects suffering HAPO revealed a decrease in DL_CO_ of more than 10% before HAPO occurred. Inter-individual response, exposure time and different altitudes may explain the unclear results in DL_CO_ modifications.

In this study, we found that pulmonary function was not negatively affected by ~15 km/day of swimming training at moderate altitude after 14 days. Swimmers trained 80 km in the pool, around 30 h per week, during the whole altitude training camp, a remarkable program at altitude in opposition to mountaineer’s expeditions. Tiller et al. [44] have also shown that pulmonary function responds well to chronic endurance exercise performed, running 10 marathons on 10 consecutive days at sea level. All the participants recruited in our study had previous experience in altitude training camps, and they also have much higher DL_CO_ basal values than predicted by their age and height, probably with very limited margin for improvement and an extraordinary capacity to face the environmental stress.

Exercise at hypoxia failed to elicit acute functional improvements in the pulmonary system in adults, suggesting that functional plasticity of the ventilatory muscles is limited in adulthood [45]. During somatic maturation, a relatively short period (5 months) of altitude exposure enhanced lung diffusing capacity for O_2_ transport and metabolic efficiency in growing dogs [33], which was maintained after return to sea level (1–2 yr) suggesting that the functional improvement during maturation may be permanent [46]. It appears that altitude exposure accentuates active lung growth during maturation but may be insufficient to reinitiate lung growth in adulthood [47]. Therefore, altitude training in young swimmers could be an interesting strategy to develop lung functional development, but in highly developed lungs, elite adult swimmers may have no physiological effect.

Despite repeated coughs and feelings of dyspnoea, our subjects did not present a limited diffusion capacity after swimming training at 1850 m of altitude. Swimming training at sea level has been associated with a decrease in DL_CO_ (under review for publication). Therefore, the acute decrease in DL_CO_ after training was expected to be aggravated after altitude exposure as an added stressor. Surprisingly, in this study, there was not a reduction in DL_CO_ after swimming training at 1850 m. Most of the swimmers presented a certain breathing discomfort induced by swimming training and/or chlorine after the last training of the altitude training camp. A breathless athlete is challenging due to a non-specific nature of symptoms [48], such as cough and wheeze and poor predictive clinical signs, and we must be cautious before relating SIPO to the athlete’s self-report of cough and breathlessness development. SIPO occurs when fluid accumulates in the lungs in the absence of water aspiration during swimming, causing acute shortness of breath and a cough productive of blood-tinged sputum [18].

Although DL_CO_ was not decreased after swimming training, there was an acute decrease in DL_CO_ (−10%) after cycling (3000 m), suggesting that 1850 m may be a safe altitude to practice endurance training but higher altitudes such as 3000 m may entail a risk for elite athletes training. The cause of the decrease of DL_CO_ after cycling is unknown, but a mild interstitial fluid accumulation may be the most suitable candidate [49]. Pulmonary capillary stress failure develops with intense exercise in healthy humans, and pulmonary vasoconstriction induced by alveolar hypoxia further augments the mechanical stress on capillary walls, leading to greater disruption of pulmonary blood–gas barrier integrity [50]. After cycling, there was also a severe decrease in RV after the combined training session (−33%), which is a compelling finding associated with a post-exercise response in altitude. Some studies have linked reductions in RV with the reversibility of obstructive lung disease [51] and the responsiveness after bronchodilator administration [52], but, to the best of our knowledge, this is the first time that a severe decrease in RV is described after exercise at high-altitude.

The most common medical problems occur at high altitudes above 2500 m [53], where a significant decrease in O_2_ transport to the tissues occurs [54]. Although lower lung volumes are one determinant of the augmented pulmonary arterial pressures at altitude and the consequent susceptibility to HAPO [55], it has been reported that exercise at altitude may produce interstitial pulmonary oedema in cyclists [50] due to a substantial increase in pulmonary capillary hydrostatic pressure [56], increasing alveolar fluid flooding [57,58]. In fact, interstitial oedema has been presented whenever microvascular filtration is increased because it is the mechanism that protects against the development of severe oedema [22]. Therefore, interstitial lung oedema could be considered as the interface between tissue repair and the manifestation of a severe disease after exercise or altitude exposure [22]. Finally, a common pathophysiologic pathway seems to be shared by SIPO and HAPO, allowing translation of preventive and therapeutic strategies for HAPO such as progressive acclimatization, avoidance of excessive exertion, and use of drugs that increase the availability of nitric oxide into the unexplored field of SIPO prevention and treatment [2].

### Strengths and Limitations

While DL_CO_ is consistent with an increase in extravascular lung water [42,59,60], a combination of indirect and direct techniques, such as sensitive lung function tests like DL_CO_, and imaging techniques such as chest imaging or pulmonary echography, would be the most suitable approach to definitively demonstrate the presence of a mild perturbation in extravascular water balance [22]. The main value of this study resides in the integration of lung function measures and the collaboration with coaches for volume and intensity assignment in a susceptible environment, such as the altitude training stage.

Previous lung diffusing capacity for carbon monoxide (DL_CO_) at sea level was not obtained, and as Martinot et al. [40] showed, there could be changes in DL_CO_ from sea level to day 3 after altitude arrival. However, the value of the data here presented relies on the analysis of conditions; milder symptoms may occur in order to reflect the true impact of SIPO in swimming [18].

This study was performed on a small sample of elite swimmers studied in their own environment under their own routines, which is very difficult to obtain. Measures of alveolar–capillary membrane conductance (D_M_) or pulmonary capillary blood volume (V_C_) were not obtained, which would have provided a deeper explanation of the changes in lung diffusion capacity. However, the changes in DL_CO_ and K_CO_ are still relevant and coherent with those in VA as to be considered as a correct evaluation of lung diffusion capacity in elite swimmers. 

## 5. Conclusions

A 14-days training camp at moderate altitude does not change lung diffusion capacity in elite swimmers, although there was a decrease in VA and an increase in K_CO,_ keeping DL_CO_ unaltered. An acute swimming session does not change lung diffusing capacity, but a posterior cycling session at normobaric-simulated 3000 m reduced DL_CO_ and RV in elite swimmers.

The high level of fitness and experience at altitude may have influenced these results. Although effect sizes oscillated in the range of small to medium effect, the number of participants in our study is rather limited. Further investigations should consider a larger sample size, with different levels of fitness and experience in altitude training, to confirm these results.

## Figures and Tables

**Table 1:** Anthropometrical and physical capacity parameters of the studied sample of elite swimmers.

Anthropometric and Spirometric Parameters (Units)	Elite Swimmers (n = 8)	
	Female (n = 5)	Male (n = 3)
Age (y)	18.2 ± 3.3	18.0 ± 1.7
Height (cm)	170.6 ± 4.7	178.3 ± 2.1
Body weight (Kg)	62.0 ± 3.9	69.0 ± 2.0
BMI	21.3 ± 0.5	21.7 ± 0.7
6 skinfolds	83.3 ± 13.5	49.3 ± 8.5
VO_2_max (mL·Kg^−1^·min^−1^)	55.8 ± 2.1	59.2 ± 8.4
V_E_max (L·min^−1^)	110.4 ± 11.3	138.6 ± 13.6
FVC (L)	4.4 ± 0.4	5.8 ± 1.0
FVC (%-predicted)	108 ± 10	114 ± 18
FEV1 (L)	3.8 ± 0.4	4.6 ± 0.8
FEV1 (%-predicted)	107 ± 7	108 ± 16
FEV1/FVC	85.2 ± 2.5	79.5 ± 1.2
PEF (L·s^−1^)	7.3 ± 0.9	8.2 ± 0.9
MEF25-75 (L·s^−1^)	4.0 ± 0.7	4.3 ± 0.7

**Table 2:** Lung capacity and pulmonary gas diffusion parameters before (day 3) and after (day 14) altitude training camp at 1850 m in elite swimmers.

Pulmonary Parameters (Units)	Elite Swimmers (n = 8)		
	Pre	Post	*p*-Value
DL_CO_ (mL·min^−1^·mmHg^−1^)	44.8 ± 12.4	45.0 ± 14.3	0.974
DL_CO_ (%-predicted)	160 ± 33	159 ± 34	
DL_CO_ adj (mL·min^−1^·mmHg^−1^)	40.4 ± 11.2	40.4 ± 12.8	0.966
DL_CO_ adj (%-predicted)	144 ± 30	143 ± 30	
K_CO_ (mL·min^−1^·mmHg^−1^·L^−1^)	6.23 ± 1.03	6.83 ± 1.31	**0.038**
K_CO_ (%-predicted)	126 ± 25	138 ± 29	
VA (L)	7.13 ± 1.61	6.50 ± 1.59	**0.005**
VA (%-predicted)	127 ± 18	116 ± 18	
TLC (L)	7.28 ± 1.61	6.65 ± 1.59	**0.005**
TLC (%-predicted)	127 ± 18	116 ± 18	
VC_IN_ (L)	4.76 ± 1.12	4.35 ± 1.52	0.130
RV (L)	2.51 ± 0.74	2.30 ± 0.57	0.381

Marginal significance (close to 0.05) in bold and italic characters.

**Table 3:** Lung capacity and pulmonary gas diffusion parameters before and after a combined session (day 10) of swimming at 1850 m in hypobaric hypoxia and cycling at 3000 m in normobaric hypoxia.

Pulmonary Parameters (Units)	Elite Swimmers (n = 8)					
	Pre	Mid	Pre vs. Mid *p*-Value	Post	Pre vs. Post *p*-Value	Mid vs. Post *p*-Value
DL_CO_ (mL·min^−1^·mmHg^−1^)	45.8 ± 14.5	45.2 ± 12.0	1.000	41.1 ± 12.8	0.156	**0.044**
DL_CO_ (%-predicted)	166 ± 30	165 ± 26		150 ± 32		
DL_CO_ adj (mL·min^−1^·mmHg^−1^)	41.1 ± 13.0	40.6 ± 10.8	1.000	36.8 ± 11.5	0.153	**0.044**
DL_CO_ adj (%-predicted)	149 ± 27	148 ± 24		134 ± 29		
K_CO_ (mL·min^−1^·mmHg^−1^·L^−1^)	6.34 ± 1.00	6.27 ± 1.16	1.000	6.17 ± 1.13	1.000	***0.053***
K_CO_ (%-predicted)	132 ± 14	134 ± 28		126 ± 23		
VA (L)	6.53 ± 1.35	6.37 ± 1.24	1.000	5.66 ± 0.52	0.330	1.000
VA (%-predicted)	125 ± 18	123 ± 14		118 ± 18		
TLC (L)	6.68 ± 1.35	6.52 ± 1.24	1.000	5.81 ± 0.52	0.330	1.000
TLC (%-predicted)	124 ± 18	123 ± 14		118 ± 17		
VC_IN_ (L)	4.69 ± 1.15	4.73 ± 1.12	1.000	4.22 ± 0.31	0.823	1.000
RV (L)	2.37 ± 0.63	2.16 ± 0.65	1.000	1.78 ± 0.59	**0.001**	0.266

Marginal significance (close to 0.05) in bold and italic characters.
